# Bone marrow-derived, alternatively activated macrophages enhance solid tumor growth and lung metastasis of mammary carcinoma cells in a Balb/C mouse orthotopic model

**DOI:** 10.1186/bcr3195

**Published:** 2012-05-22

**Authors:** Han Jin Cho, Jae In Jung, Do Young Lim, Gyoo Taik Kwon, Song Her, Jong Hoon Park, Jung Han Yoon Park

**Affiliations:** 1 Department of Food Science and Nutrition, Hallym University, Chuncheon 200-702, Korea; 2 Division of Bio-Imaging, Chuncheon Center, Korea Basic Science Institute, Chuncheon 200-701, Korea; 3 Department of Biological Science, Sookmyung Women's University, Seoul 140-742, Korea

## Abstract

**Introduction:**

Tumor-associated macrophages, which are derived from the infiltration of circulating bone marrow-derived monocytes, consist primarily of a polarized M2 macrophage (M2-Mϕ) population and are associated with poor prognosis in various cancers. In the present study, we attempted to assess whether M2-Mϕs derived from bone marrow stimulate the promotion and progression of mammary tumors.

**Methods:**

4T1 murine mammary carcinoma cells were injected either alone or coupled with M2-Mϕs into the mammary fat pads of syngeneic female Balb/C mice. M2-Mϕs were prepared by treating monocytes isolated from female Balb/C mouse bone marrow with IL-4. Tumor cell growth was determined using an *in vivo *imaging system and the expression of cell proliferation-related, angiogenesis-related, and lymphangiogenesis-related proteins in tumor tissues was immunohistochemically analyzed. To evaluate the effects of the crosstalk between 4T1 cells and M2-Mϕs on the secretion and mRNA expression of cytokines and the migration of monocytes, 4T1 cells and M2-Mϕs were co-cultured and cytokine antibody array, real-time RT-PCR, and trans-well migration assays were conducted.

**Results:**

The co-injection of M2-Mϕs into the mammary fat pads of mice increased solid tumor growth and lung metastasis of 4T1 cells as well as the infiltration of CD45^+ ^leukocytes into tumor tissues. The proportions of Ki-67^+ ^proliferating cells and the expression of hypoxia inducible factor-1α, vascular endothelial cell growth factor A, CD31, vascular endothelial cell growth factor C, and lymphatic vessel endothelial receptor-1 were increased significantly in the tumor tissues of mice co-injected with 4T1 cells and M2-Mϕs. The *in vitro *results revealed that the proliferation of 4T1 cells, the migration of monocytes, and the secretion of granulocyte colony-stimulating factor, IFNγ, IL-1α, IL-2, IL-16, IFNγ-induced protein-10, keratinocyte-derived chemokine, macrophage colony-stimulating factor, monocyte chemotactic protein-1, macrophage inflammatory protein-1α, and RANTES were increased when 4T1 cells were co-cultured with M2-Mϕs, as compared with when the 4T1 cells were cultured alone.

**Conclusion:**

The crosstalk between 4T1 cells and M2-Mϕs increased the production of cytokines, which may have induced immune cell infiltration into tumor tissues, tumor cell proliferation, angiogenesis, and lymph angiogenesis, thereby increasing solid tumor growth and lung metastasis.

## Introduction

Macrophages, which are derived from bone marrow progenitors, are recruited into tissues to replace resident populations or react to a variety of inflammatory and immune stimuli. The differentiated phenotype of recruited macrophages reflects signals from the microenvironment in which they dwell. These macrophages are broadly divided into two main classes; classically activated macrophages, or alternatively activated macrophages (M2-Mϕs). Classically activated macrophages have immunostimulatory, T-helper type 1-orienting properties and have an IL-1^high^, IL-6^high^, IL-12^high^, IL-23^high^, TNF^high^, and IL-10^low ^profile. By way of contrast, M2-Mϕs have an IL-1^low^, IL-6^low^, IL-12^low^, TNF^low^, and IL-10^high ^profile, poor antigen-presenting capacity, and are reported to suppress T-helper type 1 adaptive immunity (reviewed in [1]).

Solid tumors consist of a number of cells, including malignant cells, fibroblasts, endothelial cells, and immune cells including macrophages. As cancer cells generate chemotactic factors for monocytes, macrophage accumulations were frequently observed in a variety of cancers, including breast cancer [2-4]. Tumor microenvironments produce a variety of factors, which lead to promotion of the differentiation and polarization of infiltrated monocytes into M2-Mϕs [5]. The macrophage within the tumor, which is referred to as tumor-associated macrophage (TAM), exhibits several pro-tumoral functions, including the promotion of angiogenesis, the suppression of adaptive immunity, and the expression of growth factors and matrix proteases (reviewed in [1]). Recent evidence has shown that TAM is associated with poor prognosis in cancers, including breast cancer, lung cancer, and pancreatic cancer [2,4,6]. In a mouse model of mammary tumors initiated by the expression of the polyoma virus middle T oncoprotein, the null mutation in the colony-stimulating factor-1 gene *CSF-1 *to deplete macrophages has been demonstrated to reduce the progression of preinvasive lesions to malignant lesions and attenuate the formation of lung metastasis [7]. Using a 4T1 orthotopic Balb/C mammary cancer model in which 4T1 mammary carcinoma cells were injected into the mammary fat pads of syngeneic Balb/C mice, Luo and colleagues have shown that a legumain-based DNA vaccine targeting TAM suppressed tumor angiogenesis and metastasis [8].

In this study, we evaluated the effect of M2-Mϕs on the promotion of mammary cancer using the 4T1-orthotopic tumor model in which 4T1 mammary carcinoma cells were injected in conjunction with M2-Mϕs. The 4T1 cell line was derived from a naturally occurring mammary tumor in a Balb/C mouse [9] and thus the inoculated 4T1 cells grow into solid tumors that spontaneously metastasize to the lung, whereas the original tumor continues to grow in the mammary fat pads. The 4T1 model is thus a physiological mouse model that closely mimics breast cancer in humans [9,10]. To prepare M2-Mϕs, we isolated monocytes from the bone marrow of female Balb/C mice and treated them with IL-4. The M2-Mϕs and 4T1 cells are syngeneic in Balb/C mice, and - as opposed to immune-deficient nude mice, which are extensively used as *in vivo *tumor models - BALB/c mice are immune competent. This animal model is therefore useful in the study of tumor growth and progression, in which the involvement of the inflammatory/immune system is a critical consideration.

We used an *in vivo *imaging system to estimate the accumulation of 4T1 tumor cells in the solid tumor. To quantify tumor cells, we engineered 4T1-luc cells that stably and constitutively expressed a firefly luciferase enzyme. The bioluminescence signal is derived from viable 4T1-luc cells only, not from other cell types present in a tumor such as fibroblasts, endothelial cells, and immune cells. Additionally, the *in vivo *imaging system is useful for repeatedly estimating the growth of a solid tumor over the course of an experiment, with each animal serving as its own reference. We demonstrated that the co-injection of bone marrow-derived M2-Mϕs with 4T1 cells into mammary fat pads effectively stimulated solid tumor growth and lung metastasis.

## Materials and methods

### Antibodies

The following antibodies were purchased from the suppliers as indicated: antibodies against Ki-67, hypoxia-inducible factor-1 alpha (HIF-1α), and lymphatic vessel endothelial hyaluronan receptor-1 from Abcam (Cambridge, UK); antibodies against cyclin-dependent kinase (CDK)2, CDK4, vascular endothelial cell growth factor (VEGF)-A, VEGF-C, and CD31 from Santa Cruz Biotechnology (Santa Cruz, CA, USA); anti-CD45 antibody from R&D Systems (Minneapolis, MN, USA); antibodies against rabbit-IgG-Alexa488, rabbit-IgG-Alexa597, and goat-IgG-Alexa488 from Invitrogen (Carlsbad, CA, USA); and anti-goat-IgG-Cy3 antibody from Rockland (Gibertsville, PA, USA). The primary and secondary antibodies were used at a dilution of 1:200 and 1:1,000, respectively.

### Cell culture

The 4T1 cell line was purchased from the American Type Culture Collection (Manassas, VA, USA). To establish a 4T1 cell line that stably expresses firefly luciferase (4T1-luc), the cells were infected with a lentiviral vector harboring a gene encoding for firefly luciferase [11]. The 4T1 and 4T1-luc cell lines were maintained in DMEM containing 10% FBS. The L929 murine fibrosarcoma cell line was purchased from the Korean Cell Line Bank (Seoul, Korea) and maintained in RPMI 1640 containing 10% heat-inactivated FBS. All cultures contained 100 kU/l penicillin and 100 mg/l streptomycin.

### Preparation of M2-Mϕs derived from mouse bone marrow

Bone marrow cells were collected from the femoral shafts of female Balb/C mice. Monocytes were isolated from the bone marrow cell suspensions using the EasySep mouse monocyte negative-selection enrichment mixture (StemCell Technologies, Vancouver, Canada) in accordance with the manufacturer's protocols. The isolated monocytes were cultured and differentiated for 7 days in DMEM containing 10% heat-inactivated FBS and 10% L929 cell-conditioned medium [12]. The cell monolayers were then treated overnight with IL-4 to induce differentiation into the M2 phenotype. To determine whether the macrophages evidence the characteristics of M2-Mϕs, the expression of macrophage mannose receptors was routinely estimated via RT-PCR [13].

### Animals and *in vivo *imaging system

Female Balb/C mice (3 weeks of age) were purchased from Orient Bio Inc. (Gapyung, Korea) and were acclimatized to laboratory conditions at the animal research facility of Hallym University, Chuncheon, Korea. All animal experimental protocols were approved by the Animal Care and Use Committee of Hallym University (Hallym2010-14). Balb/C mice were fed on an AIN-76A diet (Research Diets, New Brunswick, NJ, USA) and water *ad libitum*. After acclimatization, 4T1-luc cells (5 × 10^4 ^cells) and/or M2-Mϕs (1 × 10^4 ^cells) were suspended in 0.1 ml Matrigel (BD Biosciences, San Jose, CA, USA) and injected into the inguinal mammary fat pads of female Balb/C mice (4 weeks of age, eight mice/group).

For the bioluminescence imaging, mice received an intraperitoneal injection of 150 μl D-luciferin (30 mg/ml). Fifteen minutes after the injection of D-luciferin, the mice were anesthetized with isoflurane/oxygen and placed on the imaging stage. The bioluminescence signals were monitored using an IVIS-200 (Xenogen Corp., Alameda, CA, USA). The data were analyzed using the total photon flux emission (photons/second) in the regions of interest.

Three weeks after 4T1 cell injection, mice were anesthetized and the tumors were removed and weighed. The lungs were fixed in Bouin's solution, and the metastases were quantified.

To determine whether immune cells in the tumor tissues are derived from injected M2-Mϕs or infiltrated circulating leukocytes, a separate animal experiment was conducted. For this experiment, M2-Mϕs were labeled with the green fluorescence-tagged lipophilic tracer 3,3'-dioctadecyloxacarbocyanine perchlorate/DiOC_18 _(DiO; Invitrogen) immediately prior to the injection. To accomplish this, the M2-Mϕs were exposed to 50 μmol/l DiO for 2 hours and were washed with PBS. The animals (five mice/group) were sacrificed 2 weeks after the injection.

### Immunohistochemical and immunofluorescence staining

For immunohistochemical staining, tumors were fixed in 4% paraformaldehyde and embedded in paraffin wax. Sections of 5 μm were prepared from the paraffin-embedded tumor blocks and hydrated through xylene and graded alcohol. Antigen retrieval was performed by microwaving the slides in 10 mmol/l sodium citrate buffer (pH 6.0). Endogenous peroxidases were quenched with 3% hydrogen peroxide for 5 minutes. Sections were incubated with their relevant antibodies, and were then developed using an LSAB kit (Dako, Carpinteria, CA, USA) in accordance with the manufacturer's instructions.

For immunofluorescence staining, tumors were immersed in optimal cutting temperature compound and frozen rapidly in liquid nitrogen. Then, 5 μm sections were cut with a cryostat microtome. Sections were fixed with 4% paraformaldehyde, permeabilized with ice-cold methanol, and incubated with their relevant antibodies. Nuclei were counterstained with 4',6-diamidino-2-phenylindole.

### Co-culture of 4T1 cells and M2-Mϕs

To investigate the cytokine production, 4T1 cells (1 × 10^5 ^cells/well) were plated in 12-well plates with or without M2-Mϕs (2 × 10^4 ^cells/well). After 24 hours, cells were serum starved for 24 hours with DMEM. After serum starvation, cells were incubated in fresh DMEM for a further 24 hours, and the 24-hour-conditioned media were collected. Four independent experiments were conducted and the conditioned media were pooled. The relative levels of cytokines in the pooled conditioned media were measured via a mouse cytokine array panel A kit (ARY006) in accordance with the manufacturer's instructions (R&D Systems). Densitometric analysis was conducted using the Bioprofile BioID application (Vilber Lourmat, Marne-la-Vallée, France). Expression levels were normalized to the levels of the positive control spots (contained within the membrane), and the positive control levels were set at 100%.

To determine the expression of cytokine mRNAs in a co-culture model, 4T1 cells or M2-Mϕs were plated on cell culture coverslips (24 mm × 30 mm) in eight-well plates (Nunc Inc., Rochester, NY, USA). After 24 hours, two coverslips (covered with the monolayers of 4T1 cells or M2-Mϕs) were placed in the same wells of four-well plates (4T1 + 4T1; 4T1 + M2-Mϕs; or M2-Mϕs + M2-Mϕs) and incubated in fresh DMEM for 24 hours. The total RNAs of each cell type were isolated using an RNeasy Plus Mini Kit (Qiagen, Valencia, CA, USA), and the real-time RT-PCR was conducted as previously described [14]. The sequences for PCR primer sets are listed in Table 1. The control levels (4T1 cells alone or M2-Mϕs cells alone) were set at 1.

**Table 1:** Primer sequences used for real-time RT-PCR

mRNA	Primer sequences	Reference
M-CSF	Forward: 5'-TTGGCTTGGGATGATTCTCAG-3'	[28]
	Backward: 5'-GCCCTGGGTCTGTCAGTCTC-3'	
IP-10	Forward: 5'-CCCACGTGTTGAGATCATTG-3'	[29]
	Backward: 5'-GCTCTCTGCTGTCCATCCAT-3'	
RANTES	Forward: 5'-CCCTCACCATCATCCTCACT-3 '	[30]
	Backward: 5'-CCACTTCTTCTCTGGGTTGG-3'	
MCP-1	Forward: 5'-CAGCCAGATGCAGTTAACGC-3'	
	Backward: 5'-CCTCTCTCTTGAGCTTGGTG-3'	
G-CSF	Forward: 5'-CATGGCTCAACTTTCTGCCCA-3'	[31]
	Backward: 5'-TAGGTGGCACACAACTGCTC-3'	
IL-1α	Forward: 5'-AAGACAAGCCTGTGTTGCTGAAGG-3'	[32]
	Backward: 5'-CCAGAAGAAAATGAGGTCGGTC-3'	
KC	Forward: 5'-CAAGAACATCCAGAGCTTGAAGGT-3'	[33]
	Backward: 5'-GTGGCTATGACTTCGGTTTGG-3'	
MIP-1α	Forward: 5'-ACCATGACACTCTGCAACCA-3'	[34]
	Backward: 5'-CCCAGGTCTCTTTGGAGTCA-3'	

G-CSF, granulocyte colony-stimulating factor; HIF-1α, hypoxia-inducible factor-1α; IP-10, IFNγ-induced protein-10; KC, keratinocyte chemoattractant; M-CSF, macrophage colony-stimulating factor; MCP-1, monocyte chemotactic protein-1; MIP-1α, macrophage inflammatory protein-1 alpha; RANTES, regulated upon activation, normal T-cell expressed and secreted.

### Trans-well migration assays of bone marrow-derived monocytes

Monocytes were isolated as described above and plated onto the type IV collagen-coated filter (3 μm pore size) in 6.5-mm trans-well inserts at 1 × 10^5 ^cells. The lower chamber of the well was filled with or without media conditioned by 4T1 cells and/or M2-Mϕs. Monocytes were incubated for 1 hour. Migrated cells were stained with 4',6-diamidino-2-phenylindole and were quantified by counting the 4',6-diamidino-2-phenylindole-stained cells.

### Statistical analysis

The results are expressed as the mean ± standard error of the mean. Differences between groups were assessed via the Student's *t *test and Duncan's multiple-range test, utilizing SAS statistical software, version 8.12 (SAS Institute, Cary, NC, USA).

## Results

M2-Mϕs stimulate the solid tumor growth of 4T1 mammary cancer cells in Balb/C mice

**Figure 1:**
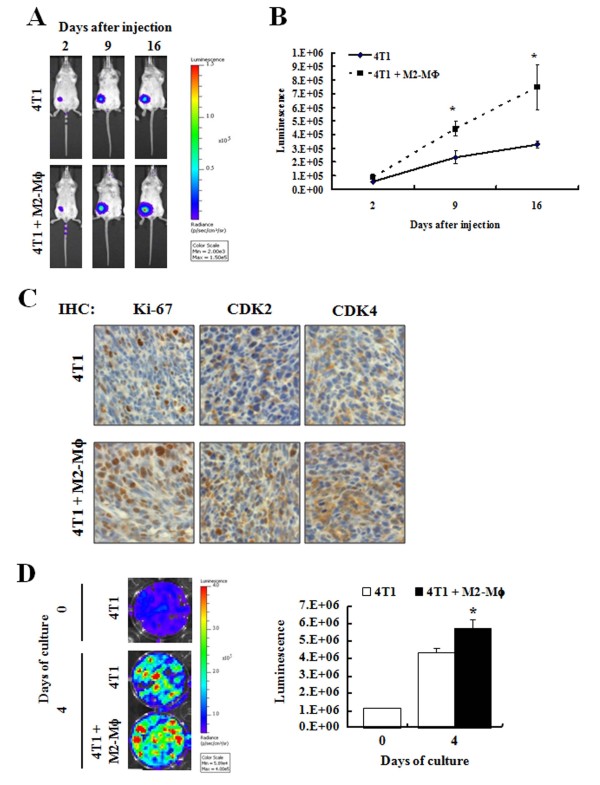
**M2-polarized macrophages enhance tumor growth and increase cell proliferation indices**. 4T1-luc cells and/or M2-polarized macrophages (M2-Mϕs) were injected into the mammary fat pads of female Balb/C mice. Bioluminescent signals were monitored on the days indicated. **(A) **Representative images of luciferase signals. **(B) **Quantitative analysis of luminescence. Each bar represents the mean ± standard error of the mean (SEM) (*n *= 8). **(C) **Immunohistochemical (IHC) staining for Ki-67, cyclin-dependent kinase (CDK)2, and CDK4 in tumor sections was based on 3,3'-diaminobenzidine staining, as described in the Materials and methods section. **(D) **4T1-luc cells and/or M2-Mϕs were cultured in 24-well plates. Bioluminescent signals were monitored at the indicated day. Each bar represents the mean ± SEM from three independent experiments performed in duplicate. *Significantly different from the 4T1 group, *P *< 0.05.

To determine whether M2-Mϕs stimulate the growth of mammary cancer cells *in vivo*, we injected 4T1-luc cells into the mammary fat pads of Balb/C either alone or coupled with M2-Mϕs. Prior to the injection, the levels of macrophage mannose receptor transcripts were estimated via RT-PCR in order to determine whether the IL-4-treated cells exhibit an M2 phenotype. IL-4 treatment effectively induced the mRNA expression of the macrophage mannose receptor (data not shown). At the indicated time point after the tumor cell inoculation, all mice were scanned with the IVIS-200 system (Xenogen Corp.) and each bioluminescent signal was calculated. At 2 days after the inoculation, there was no significant difference in the bioluminescent signals between the two groups. Co-injection with M2-Mϕs significantly increased bioluminescent signals at 9 days after the inoculation and the increase in bioluminescent signals was more pronounced at 16 days (Figure 1A,B). These results clearly demonstrate that the accumulation of 4T1 cancer cells in the mammary fat pads was increased significantly when the 4T1 cells were co-injected with M2-Mϕs as compared with when 4T1 cells were injected alone. In a different experiment (10 mice/group) we measured tumor volumes and weights, which were noted to be increased when 4T1 cells were co-injected with M2-Mϕs (data not shown).

As the tumor growth was increased in mice co-injected with 4T1 cells and M2-Mϕs, we subsequently evaluated the effects of M2-Mϕs on the expression levels of proteins involved in cell proliferation via immunohistochemistry. As compared with mice injected with 4T1 cells alone, the proportions of Ki-67-positive cells (Ki-67 is a nucleoprotein expressed only in cycling cells) and the expression of CDK2 and CDK4 were increased significantly in mice co-injected with M2-Mϕs (Figure 1C). To determine whether M2-Mϕs stimulate 4T1 cell proliferation, an *in vitro *study was conducted by culturing 4T1-luc cells alone or with M2-Mϕs. A positive correlation was noted between 4T1-luc cell numbers and bioluminescent signals (*R*^2 ^= 0.9988). Bioluminescent signals were significantly increased when the 4T1-luc cells were co-cultured for 4 days with M2-Mϕs compared with when 4T1-luc cells were cultured alone, indicating that the crosstalk between the two cell types induces the proliferation of 4T1 cells (Figure 1D).

### M2-Mϕs induce an increase in lung metastasis of 4T1 mammary cancer cells in Balb/C mice

**Figure 2:**
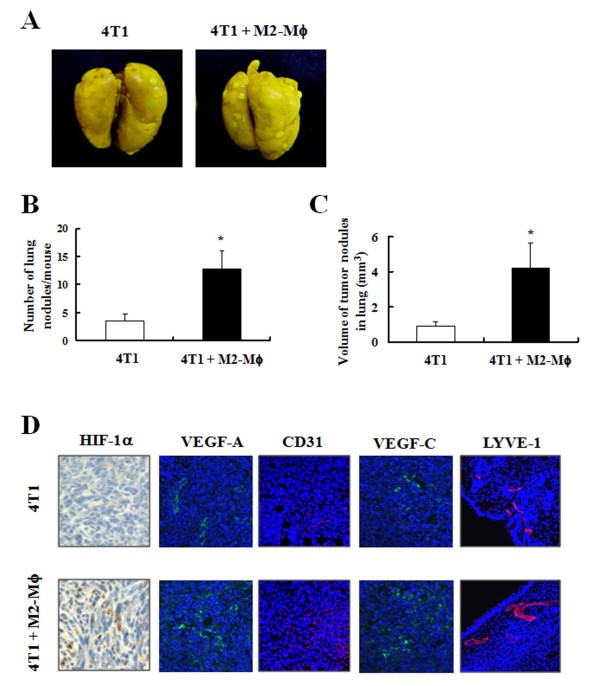
**M2-polarized macrophages enhance lung metastasis of 4T1 cells in the 4T1 orthotopic mouse tumor model**. 4T1 cells and/or M2-polarized macrophages (M2-Mϕs) were injected into the mammary fat pads of female Balb/C mice. After 3 weeks, the mice were killed and the lungs were removed and fixed in Bouin's solution. **(A) **Photographs of lung metastasis. **(B), (C) **Quantitative analysis of lung tumor nodules. Each bar represents the mean ± standard error of the mean (*n *= 8). *Significantly different from the group injected with 4T1 cells alone, *P *< 0.05. **(D) **Tumor sections were stained with their relevant antibodies as described in the Materials and methods section. Photographs of immunohistochemical or immunofluorescent staining, which are representative of six different animals, are shown. HIF-1α, hypoxia-inducible factor-1 alpha; VEGF, vascular endothelial growth factor; LYVE-1, lymphatic vessel endothelial hyaluronan receptor-1.

To assess the effect of M2-Mϕs on the lung metastasis of mammary cancer cells, 4T1-cell-bearing mice were sacrificed at 3 weeks after the inoculation, the lungs were fixed in Bouin's solution, and the metastases were quantified. At this time, tumor nodules were detected in all animals injected with 4T1 cells. The number and volume of lung tumor nodules per mouse were increased significantly in animals co-injected with M2-Mϕs (Figure 2A to 2C).

HIF-1α activates the transcription of multiple genes including VEGF-A, a key factor in tumor angiogenesis. The accumulation of HIF-1α in the nuclei of tumor cells was increased in mice co-injected with 4T1 cells and M2-Mϕs (Figure 2D). Co-inoculation of M2-Mϕs increased the expression of VEGF-A as well as the proportions of CD31-positive cells (CD31 is a glycoprotein expressed on endothelial cells and in platelets) (Figure 2D). Additionally, the co-inoculation of M2-Mϕs increased the expression of the lymphangiogenic growth factor VEGF-C and lymphatic vessel endothelial hyaluronan receptor-1-positive vessels.

### Crosstalk between 4T1 cells and M2-Mϕs increases the infiltration of monocytes into tumor tissues in Balb/C mice

**Figure 3:**
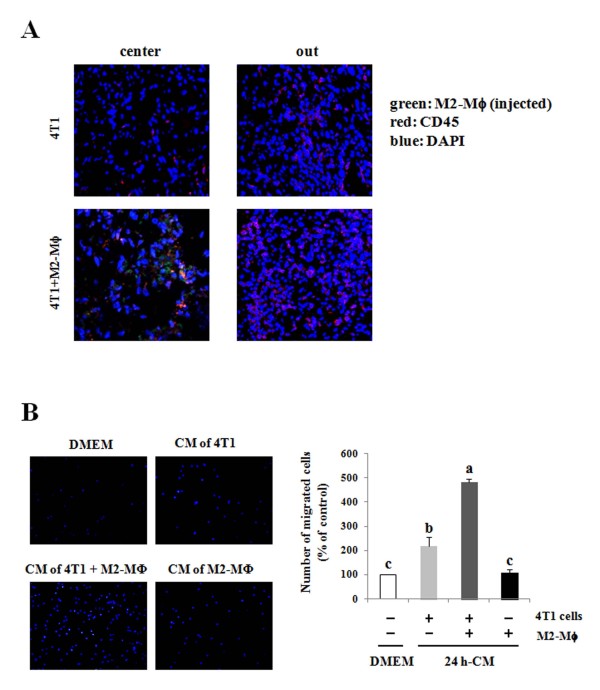
**M2-polarized macrophages enhance leukocyte infiltration into tumor tissues and monocyte migration *in vitro***. **(A) **4T1 cells and/or M2-polarized macrophages (M2-Mϕs) were injected into the mammary fat pad of female Balb/C mice. M2-Mϕs were labeled with 3,3'-dioctadecyloxacarbocyanine perchlorate/DiOC_18 _(DiO) (green) prior to the injection. Cryostat sections of tumor were stained with anti-CD45 antibody (red), and nuclei were counterstained with 4',6-diamidino-2-phenylindole (DAPI) (blue). **(B) **4T1 cells and/or M2-Mϕs were plated in 12-well plates, and 24-hour-conditioned media (CM) were collected for trans-well migration assays. The migration of bone marrow-derived monocytes was estimated using the CM as a chemoattractant. The migrated monocytes were quantified by counting the DAPI-stained cells. Each bar represents the mean ± standard error of the mean of three independent experiments performed in duplicate. Means without the same lowercase letter differ, *P *< 0.05.

In an effort to determine whether the co-injection of M2-Mϕs induces the infiltration of circulating monocytes into tumor tissues, M2-Mϕs were labeled with the green fluorescence-tagged, lipophilic tracer DiO immediately before being injected. At 2 weeks after the tumor cell injection, the injected M2-Mϕs (green color) were observed only in the central area of the tumor. Tumor sections were stained with a red fluorescence-tagged CD45 antibody. CD45^+ ^cells (red color) were distributed diffusely throughout the tumor area and increased in mice co-injected with 4T1 cells and M2-Mϕs (Figure 3A). Additionally, *in vitro *trans-well migration assay results revealed that the migration of bone marrow-derived monocytes was tremendously elevated when medium conditioned by 4T1/M2-Mϕ co-cultures was used as a chemoattractant, as compared with medium conditioned by 4T1 cells or M2-Mϕs alone (Figure 3B).

### Crosstalk between 4T1 cells and M2-Mϕs stimulates cytokine production

**Figure 4:**
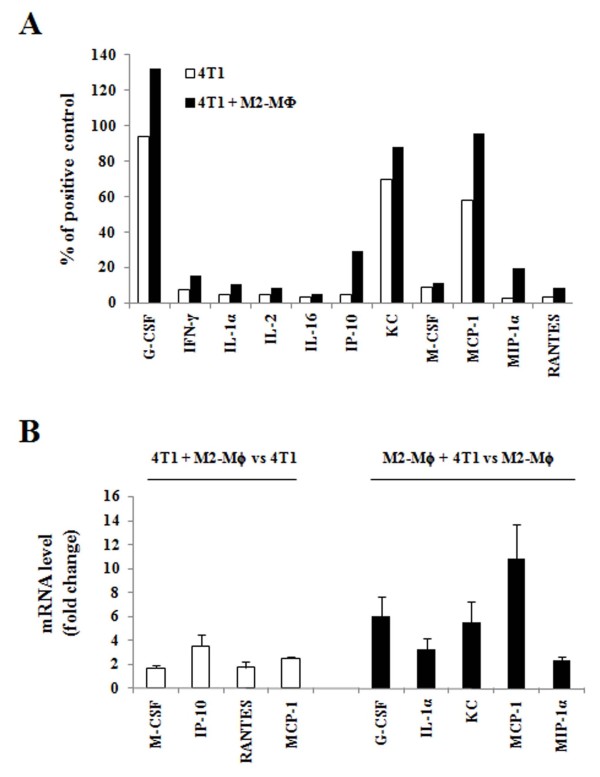
**Crosstalk between 4T1 cells and M2-polarized macrophages stimulates cytokine production**. **(A**) 4T1 cells and/or M2-polarized macrophages (M2-Mϕs) were plated in 12-well plates, and 24-hour-conditioned media (CM) were collected. Four independent experiments were conducted and the CM were pooled. The relative levels of cytokines in the pooled CM were estimated using a mouse cytokine array panel A kit. Expression levels were normalized to the levels of the positive control spots (contained within the membrane), and the positive control levels were set at 100%. **(B) **4T1 cells or M2-Mϕs were plated on cell culture coverslips (24 mm × 30 mm) in eight-well plates. After 24 hours, coverslips (containing 4T1 cells or M2-Mϕs) were placed on the same wells of four-well plates (4T1 + 4T1; 4T1 + M2-Mϕs; or M2-Mϕs + M2-Mϕs) for 24 hours. Total RNA was isolated, and real-time RT-PCR was performed. Results are shown as means ± standard error of the mean of three independent experiments performed in duplicate. Each of the control levels (4T1 cells alone or M2-Mϕs alone) was set to 1. G-CSF, granulocyte colony-stimulating factor; IP-10, IFNγ-induced protein-10; KC, keratinocyte chemoattractant; M-CSF, macrophage colony-stimulating factor; MCP-1, monocyte chemotactic protein-1; MIP-1α, macrophage inflammatory protein-1 alpha; RANTES, regulated upon activation, normal T-cell expressed and secreted.

The results of the mouse cytokine antibody array analyses indicated that the levels of granulocyte colony-stimulating factor (G-CSF), IFNγ, IL-1α, IL-2, IL-16, IFNγ-induced protein-10 (IP-10), keratinocyte-derived chemokine **(**KC), macrophage colony-stimulating factor (M-CSF), monocyte chemotactic protein-1 (MCP-1), macrophage inflammatory protein-1α (MIP-1α), and regulated upon activation, normal T-cell expressed and secreted (RANTES) were increased in the media conditioned by the co-culture of 4T1 cells and M2-Mϕs (Figure 4A). The results of real-time RT-PCR demonstrated that the expression of M-CSF, IP-10, and RANTES mRNAs increased in 4T1 cells when 4T1 cells were co-cultured with M2-Mϕs as compared with when 4T1 cells were cultured alone. In M2-Mϕs, the mRNA expression of G-CSF, IL-1α, KC, and MIP-1α were increased when M2-Mϕs were co-cultured with 4T1 cells as compared with when M2-Mϕs cells were cultured alone. The expression of MCP-1 mRNA increased in both 4T1 cells and M2-Mϕs upon co-culture (Figure 4B). These results demonstrate that interactions between 4T1 cells and M2-Mϕs increase the production of a variety of chemoattractants, resulting in increases in tumor cell proliferation and monocyte migration.

## Discussion

The tumor microenvironment consists of several components, including malignant cells, stromal cells, extracellular matrix, endothelial cells, and infiltrating leukocytes. TAM is frequently found to constitute a major part of the leukocyte infiltrates present in the tumor microenvironment (reviewed in [1]). Extensive infiltration of TAM has been shown to be positively associated with poor prognosis in breast cancer (reviewed in [15]) and high numbers of TAMs correlate with increased tumor angiogenesis and involvement of local lymph nodes in breast cancer patients [16,17]. Emerging evidence indicates that, in the tumor microenvironment, tumor cells affect the differentiation and functional orientation of TAM. In turn, TAMs function as a key regulator in the tumor microenvironment and evidence several characteristics of M2-Mϕs: the suppression of inflammatory responses and adaptive immunity, the repair and modeling of wounded and damaged tissues, and the promotion of angiogenesis (reviewed in [18]). However, there is evidence showing that the phenotype of TAMs is pliable and varies among tumor types, stages of tumor development, and the location of TAMs in the tumor microenvironment (that is, the response of TAMs to local signals) (reviewed in [19]). Understanding the roles of the specific phenotype of macrophages on tumor promotion and tumor progression is therefore important. In the present study, we demonstrated that the co-injection of M2-Mϕs with mammary carcinoma cells into the mammary fat pad of syngeneic Balb/C mice increased solid tumor growth and lung metastasis.

In the present study, the injected green M2-Mϕs were confined to the center of the tumor tissues, whereas the CD45^+ ^leukocytes were detected diffusely throughout the tumor tissues and increased in animals co-injected with M2-Mϕs as compared with those in mice injected with 4T1 cells alone (Figure 3A). Our results indicate that the injected macrophages were not proliferating in the tumors. Indeed, in a preliminary experiment, when M2-Mϕs were injected alone, we were unable to detect any solid mass 3 weeks after the injection when the mice were sacrificed. These results demonstrate that the crosstalk between tumor cells and M2-Mϕs increases the production of chemoattractants for the infiltration of circulating leukocytes. Additionally, the media conditioned by co-culture of 4T1 and M2-Mϕs profoundly induced the migration of bone marrow-derived monocytes (Figure 3B). The co-culture conditioned media contained higher concentrations of G-CSF, IFNγ, IL-1α, IL-2, IL-16, IP-10, KC, M-CSF, MCP-1, MIP-1α, and RANTES as compared with media conditioned by 4T1 cells alone (Figure 4A). These cytokines may have induced the infiltration of circulating monocytes into the tumor tissues and/or the stimulation of mammary cancer cell proliferation and metastasis in these mice. The ability of MCP-1, MIP-1α, and RANTES to induce the migration of human blood monocytes has been reported previously [20]. Additionally, we have previously demonstrated that M-CSF stimulated the adhesion and migration of 4T1 cells under *in vitro *conditions [21]. The crosstalk between the two types of cells induced the mRNA expression of M-CSF, IP-10, RANTES, and MCP-1 in 4T1 cells and that of G-CSF, IL-1α, KC, MCP-1, and MIP-1α in M2-Mϕs (Figure 4B). Recently, Kim and colleagues reported that the conditioned medium of 4T1 cells induced the release of IL-6 and TNFα in bone marrow-derived macrophages [22]. In the media conditioned by 4T1 cells and a 4T1/M2-Mϕ co-culture, IL-6 was not detected and the co-culture induced no increase in the concentration of TNFα (data not shown). The differences between the two studies are probably attributable to differences in experimental conditions. In this study, we used a co-culture of 4T1 and IL-4-treated M2-Mϕs, whereas Kim and colleagues cultured bone marrow-derived macrophages in media conditioned by 4T1 cells [22]. Future studies will be required to delineate specifically which cytokines/chemokines are responsible for the increases in the proliferation of 4T1 cells and the infiltration of circulating monocytes into tumor tissues.

Immunohistochemical staining of tumor sections revealed that the proportions of Ki-67-positive cells and the expression of CDK2 and CDK4 were increased in mice co-injected with 4T1 cells and M2-Mϕs (Figure 1C). Moreover, the accumulation of bioluminescent signal of 4T1-luc cells was tremendously more pronounced in mice co-injected with M2-Mϕs (Figure 1A,B). Furthermore, *in vitro *co-cultures of these two cell types revealed that 4T1 cell growth was increased in the presence of M2-Mϕs (Figure 1D). The mammalian cell cycle is divided into four separate phases - the G1 phase, the S phase, the G2 phase, and the M phase - and transit through the cell cycle depends on sequential activation of CDKs. Activation of CDK4 appears to govern exit from the G0 phase and entry into an early G1 phase, and activation of CDK2 regulates transition into and passage through the S phase [23]. Overexpression of CDK2 and CDK4 was associated with breast cancer progression [24]. Taken together, the present results demonstrate that the crosstalk between 4T1 and M2-Mϕs stimulates cell cycle progression of 4T1 cells, which is probably mediated, at least in part, via the increased expression of CDK2 and CDK4. Future studies will be required to determine which cytokines are responsible for the increased growth of tumor cells.

As a tumor grows, it requires increased amounts of oxygen, resulting in hypoxia. In response to hypoxia, HIF-1α accumulates and is translocated into the nucleus, where it activates the transcription of multiple genes. Additionally, HIF-1α synthesis is regulated via the activation of the phosphatidylinositol 3-kinase or mitogen-activated protein kinase pathways in response to growth factors and cytokines (reviewed in [25]). HIF-1α leads to the upregulation of genes involved in certain aspects of cancer progression, including angiogenesis and metastasis. VEGF-A, which is known as an important mediator of tumor angiogenesis, is one of the targets of HIF-1α. Recently, several studies have demonstrated that the expression of HIF-1α correlates with that of VEGF-C, a lymphangiogenesis mediator [26,27]. We noted that the accumulation of HIF-1α in the nuclei of tumor cells and the expression of VEGF-A and VEGF-C were increased in mice co-injected with 4T1 cells and M2-Mϕs (Figure 2D). It was reported in the 4T1 syngeneic mammary cancer model that organ-specific (lung, spleen, liver, bone, and kidney) metastasis depends on the duration of tumor implant, and only lung metastasis was observed at 3 weeks after the inoculation of 4T1 cells [10]. We also noted tumor nodules in the lung at 3 weeks after the 4T1 cell injection. The number and volume of lung tumor nodules were increased in mice co-injected with 4T1 cells and M2-Mϕs (Figure 2A to 2C). The increased expression of CD31 and lymphatic vessel endothelial hyaluronan receptor-1 in tumor tissues (Figure 2D) indicates that tumor angiogenesis and lymphangiogenesis were increased in mice co-injected with M2-Mϕs. These increases in angiogenesis and lymphangiogenesis may, in turn, have contributed to increased solid tumor growth and lung metastasis in animals co-injected with M2-Mϕs.

## Conclusion

We demonstrated that when M2-Mϕs were co-injected with mammary cancer cells into the mammary fat pads of Balb/C mice, solid tumor growth and lung metastasis were increased. The proliferation of cancer cells, the infiltration of blood leukocytes into tumor tissues, and activation of HIF-1α were increased, in addition to angiogenesis and lymphangiogenesis in the tumor tissues when cancer cells were co-injected with M2-Mϕs. Our results indicate that the cross-talk between cancer cells and M2-Mϕs increased the production of chemoattractants and growth factors, which stimulated the infiltration of blood monocytes into tumor tissues and the proliferation of cancer cells, thereby increasing HIF-1α activation. The activated HIF-1α may also have induced the transcription of many genes involved in angiogenesis and lymphangiogenesis.

## Abbreviations

CD31: platelet endothelial cell adhesion molecule-1; CDK: cyclin-dependent kinase; DiO: 3,3'-dioctadecyloxacarbocyanine perchlorate/DiOC_18_; DMEM: Dulbecco's modified Eagle's medium; FBS: fetal bovine serum; G-CSF: granulocyte colony-stimulating factor; HIF-1α: hypoxia-inducible factor-1 alpha; IFN: interferon; IL: interleukin; IP-10: IFNγ-induced protein-10; KC: keratinocyte chemoattractant; M2-Mϕ: M2-polarized macrophage; M-CSF: macrophage colony-stimulating factor; MCP-1: monocyte chemotactic protein-1; MIP-1α: macrophage inflammatory protein-1α; PBS: phosphate-buffered saline; PCR: polymerase chain reaction; RANTES: regulated upon activation: normal T-cell expressed and secreted; RT: reverse transcription; TAM: tumor-associated macrophage; TNF: tumor necrosis factor; VEGF: vascular endothelial growth factor.

## Competing interests

The authors declare that they have no competing interests.

## Authors' contributions

HJC participated in the design, execution, analysis and interpretation of all results, and drafting of the manuscript. JIJ and SH participated in the design, execution, analyses, and interpretation of *in vivo *studies and helped to draft the manuscript. DYL and GTK participated in the design, execution, analyses, and interpretation of *in vitro *studies and helped to draft the manuscript. JHP participated in data interpretation and coordination of the study, and helped to draft and revise the manuscript. JHYP planned and designed this research, and drafted and revised the manuscript. All authors read and approved the final manuscript.
